# The Effects of Dairy Product Label Information on Cognition of Consumers: The Case of the China Choices

**DOI:** 10.1155/2021/5589710

**Published:** 2021-07-02

**Authors:** Ce Xu, Can Liu, Jingmin Cheng

**Affiliations:** School of Management, Shanxi Medical University, Taiyuan, Shanxi 030001, China

## Abstract

Food safety issues were a growing concern in most countries; affecting the people's health, social stability, economic development, and dairy product safety had always been an important topic of concern for consumers. This study is aimed at investigating the concerns of consumers about dairy product label information and its influencing factors. These survey data were reported for 4408 respondents with a total response rate of 96.35%. Findings revealed that consumers' concern on dairy product label information was relatively high on the whole, and there were significant differences in the level of consumers' concern on dairy product label information (*P* < 0.05). Regression results indicated that education significantly influenced consumers' concern about dairy product label information than age and Engel's coefficient (*P* < 0.05). Findings from this study will provide references for the government to disseminate dairy product knowledge to the public effectively.

## 1. Introduction

Food safety issues are a growing concern in most of the world because it is vital to people's health, economic development, social stability, and the form of the country and the government [1], and dairy product safety had always been an important topic of concern for consumers. A series of food safety incidents in China, such as Fuyang inferior milk powder in 2004, Sanlu milk powder in 2008, Fonterra “Botox bacillus” in 2013 and “Huishan high calcium milk” in 2015, had made consumers lose confidence in the quality and safety of dairy products and also had a certain effect on consumers' physical and mental health. In order to reshape the confidence of the consumer and obtain quality-assured dairy products, the government and relevant agencies tend to strictly standardize the regulation of the dairy industry. In particular, the label information on dairy products was strictly standardized [2]. Because food label information covered the main information of the food supply chain, it reflected the reality of food quality to a certain extent. Therefore, it was essential for consumers to pay more attention to food label information of food safety knowledge [3]. To improve the health level of consumers, the government and relevant agencies organized the public to participate actively in seminars or forums on food safety knowledge, so that consumers could further understand food label information, and then made them focus on the food label information. This paper is structured as follows. The next section provides a review and analysis of the literature on food label research, particularly dairy labelling. The research materials and methods of this study are then presented. The results and discussion of the results are reported in the third section, and the conclusions are discussed in the final part.

### 1.1. Literature Review

Food label is the link between food producers or operators and consumers, reducing information asymmetry to a certain extent, which is one of the most important factors influencing consumers' purchasing behaviour [4]. Also, the researchers found that all mandatory food labels were considered important and directly related to food quality and safety. Consumers' WTP for irradiated foods is influenced by label handling instructions [5]. Similarly, in research on fish, it was found that eco-labelling increased consumers' awareness of the impact of their purchasing decisions [6]. A study of the wine market found that Chinese consumers are most concerned about the content of labels in terms of origin and vintage [7]. Kim et al. [8] argue that having an organic label on a package positively influenced consumers' purchase decisions. Obayelu et al. [9] assesses consumers' perceptions and attitudes towards labeled and certified moringa products (tea, spice, and oil) and also identified factors influencing their willingness to pay for these products in Ogun State, Nigeria. Meanwhile, Lewis et al. [10] examined German and British consumers' willingness to pay for American, Canadian, and British beef, hormone-free beef production, and a gourmet label. In addition, a study reports that front-of-package labels have the potential to help consumers understand the nutrient quality of products and influence food choices [11]. It is also worth noting that food labelling is likely to play a more significant role for specific consumer groups, such as athletes or consumers who are concerned about healthy lifestyles [4].

Research on dairy labels shows that Canadian consumers prefer to buy domestic products when compared to imported dairy products and may be willing to pay a premium in some cases [12]. Another study shows that date labels play an important role in dairy waste and disposal [13].

The analysis revealed that there is not a great deal of research on dairy product label information, especially in China. With the aim to fill this gap, this study looks at the factors that influence Chinese consumers' identification and attention to dairy product labels.

## 2. Materials and Methods

### 2.1. Sampling

A stratified random cluster sampling method was used in this study. In the first stage, China was divided into six regions: North area, Northeast, Northwest, East area, South area, and Southwest; ten counties (or district, county, etc.) were randomly selected in each region. In the second stage, 70-75 people were selected from each county (region, city) for a questionnaire survey according to the scale of the local rural and urban population in the 60 sample counties, districts, or county-level cities. This survey was performed from December 2019 to March 2020 with the 4575 contacted individuals, 4408 completed the questionnaires, and the response rate was 96.35%.

### 2.2. Questionnaire Design

The content of the questionnaires was divided into two parts. The first part was the consumers' demographic characteristics, which included the consumers' gender, age, marital status, education, permanent residence, occupation, per capita income in a family, and Engel's coefficient. The second part made a noteworthy investigation of Consumers' Concerns about Dairy Product Label Information. According to China's Food Safety Law, food label information included seven items: the product even on a date and shelf life, storage conditions, production process, nutritional facts, product ingredient list, certification mark, origin, and manufacturers. The answers were rated using a 5-point Likert-type scale, ranging from 1 (not concerned) to 5 (very concerned). The research group trained more than 200 investigators on-the-spot and distributed questionnaires by stratified random cluster sampling. According to the proportion of the urban and rural population in the sample provinces and cities, the investigators would select the respondents and carried out the recovery and examination of questionnaires on the spot. If there were any mistakes or omissions, the respondents would be verified again. This study only analyzed the relevant data of consumers' concern on dairy product label information.

Two experts from the food safety field examined the validity of the final questionnaire. For the final questionnaire, Cronbach's *α* for consumers' concern on dairy product label information was 0.83. It was indicated that the internal consistency of this questionnaire was high in this study.

### 2.3. Statistical Analysis

Descriptive statistics, including frequencies and percentages, were used to summarize responses and provide a general summary of respondents' demographic characteristics. A *t*-test and one-way ANOVA were performed to analyze the differences between consumers' demographic characteristics and their concern on dairy product label information. Multiple stepwise linear regression analysis was performed to analyze consumers' concerns about dairy product label information. A *P* value of less than 0.05 was considered to be statistically significant. All statistical analyses were performed using SPSS for Windows (Version 19.0; SSPS Inc., Chicago, IL, USA).

## 3. Results and Discussion

Table 1 shows the demographic characteristics of the respondents. Of the 4408 respondents, 2149 were males (48.75%) and 2259 were females (51.25%). Basically, the proportion of male respondents was similar to that of female respondents. Most of the respondents were under age 30 (62.82%), and 52.45% of the respondents were unmarried. Most of the respondents had graduated from a university or junior college (44.51%). And 56.6% of the respondents lived in towns and cities, of which the majority of respondents were students (38.79%): 1715 people (38.91%) who had per capita income in a family of 1501-4000 RMB and 1416 people (32.12%) whose family Engel's coefficient was 40%-49%.

### 3.1. Univariate Findings

From Table 2, different demographic characteristics of consumers influenced their concern on dairy product label information. The overall score of consumers' concern on dairy product label information was 25.83 ± 5.356; the dimensional scores were 4.26 ± 0.961 for production date and shelf life, 3.86 ± 1.058 for storage condition, 3.67 ± 1.127 for the production process, 3.63 ± 1.086 for nutrition facts, 3.55 ± 1.077 for product ingredients, 3.44 ± 1.149 for a certification mark, and 3.42 ± 1.139 for the region of origin and manufacturers. The current results showed that consumers were generally concerned about the safety issue of dairy products. They were primarily more concerned about the production date and shelf life, storage conditions of dairy products than the production process, nutrition facts, product ingredient list, certification label, origin, and manufacturer of dairy product label information. Because of the production date and shelf life, dairy products' storage conditions must be indicated in the food labels [14]. Also, they played an important role in the quality and safety of the food and the purchase decision of consumers. However, essential reasons of consumers did not concern the production process, nutrition facts, product ingredient list, certification mark, origin, and manufacturer which may be attributed to the following aspects. First, the production process of dairy products was almost identical. Besides, the nutrition facts and the product ingredient list involved too much professional knowledge, and consumers could not deeply understand and judge them [15, 16]. Second, consumers could not care profoundly about the knowledge contained in the certification mark information, and they worried that the third party might be bought by some enterprises [15, 17]. Third, the storage conditions could represent the origin and manufacturer's information to a certain extent [18], so the consumers also like to be equipped with this information. The manufacturer's information about the situation, government, and relevant agencies should take appropriate measures to strengthen consumers' learning of professional knowledge of dairy product label information, such as production processes, nutrition facts, and product ingredient tables, to have in-depth comprehension and judgment of them [19].

A further comparison that the total scores of consumers' different demographic characteristics influenced their concern on dairy product label information found that different ages, marital status, education, permanent residence, occupation, per capita income in a family, and Engel's coefficient were statistically significantly different in total scores of consumers' concern on dairy product label information (*P* < 0.05). Among dairy product label information, the production date and shelf life, storage condition, nutrition facts, and product ingredient list were statistically significantly different in per capita income in a family by the comparison of groups (*P* < 0.05). In particular, consumers who had per capita income in a 6501-10000 RMB family were more concerned about dairy product label information (*P* < 0.05).

This study found that consumers' personal characteristic factors, such as age, education, and per capita income in a family, had a significant influence on consumers' concern on dairy product label information (*P* < 0.05) [20]. At present, the more concerned people about the label information of dairy products were younger and had a higher educational level [21]. Furthermore, the results indicated that young people with a higher educational level would pay more attention to dairy product safety issues. By comparing the production date and shelf life, storage conditions, nutrient facts, and product ingredient list, it was known that consumers who had per capita income in a family of 6501-10000 RMB were highly concerned about them (*P* < 0.05). This also reflected that consumers who had a higher income were more concerned about food label information in China. In particular, after the melamine milk powder incident, more and more people paid more attention to dairy product safety issues [22], and they tended to judge them by dairy product label information such as production date and shelf life and storage conditions of dairy products. Moreover, this was a particularly prominent phenomenon in per capita income in a family [23, 24]. Therefore, consumers needed to strengthen the popularization of dairy product label information, especially those who had lower academic qualifications and lower income and who were middle and older adults; meanwhile, the relevant knowledge of dairy products should be fairly and objectively publicized and popularized for low-educated and middle and older adults by the use of vivid language and visual images, with the help of media such as newspapers, radio, and television, to gradually increase the level of consumers' concern on dairy product label information. Moreover, food safety education and food subsidies should also be strengthened for those people who had a lower income. Many efforts would be made to promote the important value of the people's concern about dairy product label information and improve their concern about dairy product label information.

### 3.2. Multivariate Findings

Table 3 compares the influencing factors of consumers' concern on dairy product label information. Demographic variables were defined as independent variable *X* and dependent variable *Y* in the total score of consumers' concern on dairy product label information to perform multiple stepwise linear regression analysis. The inclusion criterion was 0.05, and the exclusion criterion was 0.10. The age, education, and Engel's coefficient were finally selected into the regression model. The results showed that education (∣*β*′ | = 0.068, *P* < 0.05) had a significant influence on consumers' concern on dairy product label information than that of age (∣*β*′ | = 0.066, *P* < 0.05) and Engel's coefficient (∣*β*′ | = 0.045, *P* < 0.05). The main factors that affected consumers' concern about dairy product label information were education, age, and Engel's coefficient. In a sense, consumers would pay more attention to dairy product label information, improve consumers' education, decrease age, and reduce Engel's coefficient.

This study showed that education was the primary factor influencing consumers' concern about dairy product label information. The consumers' concern on dairy product label information increased with the improvement of educational level [25]. To a certain extent, different levels of education also reflected consumers' purchase decisions, and it was the manifestation of health literacy level [26]. Consumers paid more attention to the quality of life, with the improvement of their level of education, and they would be more interested in dairy products with higher nutritional value. Besides, they often could acquire some information and actively learn relevant info education level, the Internet, and other channels [27, 28]. Therefore, they would pay more attention to dairy product label information. Although age and Engel's coefficient were secondary factors that influenced consumers' concern about dairy product label information, they still played an essential role in their concern about dairy product label information. Most of the people who had a more significant concern on dairy product label information were younger, and their family Engel's coefficient was lower. Furthermore, they usually had an excellent education level and an excellent professional background and demanded a higher quality of life than others. They also had some more substantial capability to understand food quality and safety [21, 29]. They would pay more attention to the food with higher nutritional value; in particular, dairy products were regularly purchased in the malls and supermarkets. And then, they would also pay more attention to dairy product label information under certain circumstances.

## 4. Conclusion

This study showed that the education level was the most important demographic influence on the level of interest in dairy labelling information, while age, household income, and Engel's coefficient were also statistically significant. The date of manufacture, shelf life, and storage conditions were the most important items of concern on the dairy product labels. This work provides some regulatory and political significance, and national authorities need to invest more in dairy education and communication so that consumers, including more diverse groups, have the right knowledge to apply to dairy shopping. It was more important for the government to improve the ability of food safety risk management, and it was the key content to help the public judge the food safety status and make the correct purchase decision [30].

This study had some limitations. This study is based on Chinese consumers; therefore, the general usefulness of the findings for other regions or countries is questionable. Also, the inconsistency of dairy product label policies in different countries may have affected the results to some extent. The data in this study are self-reported, and therefore, the presence of recall bias is possible. Considering these limitations, further cross-country studies should be conducted to understand consumer awareness and concerns about labelling of dairy products in different countries or regions. Further research should be conducted to explore how to effectively improve consumers' knowledge on the subject. In addition, the commercial use of the food label is becoming increasingly important, and conducting more case studies could help dairy companies establish a competitive advantage through the product label.

## Figures and Tables

**Table 1:** Demographic characteristics of the respondents (*n* = 4408).

Variables	Category	No.	Percentage (%)
Sex	Male	2149	48.75
	Female	2259	51.25

Age (y)	≤20	1503	34.10
	21~30	1266	28.72
	31~40	1093	24.86
	41~50	698	15.83
	51~60	179	4.06
	>60	86	1.95

Marital status	Unmarried	2312	52.45
	Married	1959	44.44
	Divorced	101	2.29
	Widowed	36	0.82

Education	≤Junior high school	974	22.10
	High school or secondary school	1379	31.28
	University or college	1962	44.51
	≥Postgraduate	93	2.11

Permanent residence	Town	2495	56.60
	Rural	1913	43.40

Occupation	Unemployed	1611	36.55
	Worker	1087	24.66
	Student	1710	38.79

Per capita income in a family (RMB)	≤500	412	9.35
	501~1500	997	22.62
	1501~4000	1715	38.91
	4001~6500	804	18.24
	6501~10000	341	7.74
	>10000	139	3.15

Engel's coefficient (%)^a^	≥60	299	6.78
	50~59	764	17.33
	40~49	1416	32.12
	30~39	1212	27.50
	<30	717	16.27

^a^Engel's coefficient was the proportion of the household's total expenditure on food purchases.

**Table 2:** Univariate analysis on the consumers' concern on dairy product label information.

Variables	Category	Production process	Product ingredients	Nutrition facts	Certification mark	Origin and manufacturers	Production date and shelf life	Storage condition	Total score	*F* or *t* value	*P* value
Sex	Male	3.70 ± 1.131	3.55 ± 1.086	3.62 ± 1.102	3.46 ± 1.143	3.43 ± 1.142	4.23 ± 0.968	3.82 ± 1.069	25.81 ± 5.482	-0.158	0.875
	Female	3.64 ± 1.122	3.54 ± 1.069	3.64 ± 1.070	3.42 ± 1.155	3.41 ± 1.137	4.30 ± 0.952	3.89 ± 1.047	25.84 ± 5.236		
	*F* or *t* value	1.920	0.200	-0.509	1.043	0.674	-2.404	-2.203			
	*P* value	0.055	0.842	0.611	0.297	0.501	0.016	0.028			

Age (y)	≤20	3.72 ± 1.121	3.58 ± 1.090	3.67 ± 1.094	3.52 ± 1.147	3.43 ± 1.150	4.28 ± 0.963	3.86 ± 1.057	26.07 ± 5.435	7.449	<0.001
	21~30	3.78 ± 1.108	3.63 ± 1.026	3.71 ± 1.038	3.51 ± 1.119	3.45 ± 1.129	4.26 ± 0.960	3.91 ± 1.036	26.25 ± 5.086		
	31~40	3.61 ± 1.095	3.47 ± 1.077	3.55 ± 1.096	3.38 ± 1.109	3.47 ± 1.104	4.22 ± 0.939	3.85 ± 1.062	25.55 ± 5.294		
	41~50	3.52 ± 1.137	3.50 ± 1.099	3.54 ± 1.108	3.28 ± 1.208	3.33 ± 1.154	4.32 ± 0.940	3.84 ± 1.056	25.32 ± 5.336		
	51~60	3.43 ± 1.156	3.37 ± 1.076	3.48 ± 1.083	3.25 ± 1.178	3.34 ± 1.176	4.09 ± 1.042	3.73 ± 1.144	24.70 ± 6.033		
	>60	3.23 ± 1.280	3.17 ± 1.267	3.35 ± 1.225	3.13 ± 1.156	3.21 ± 1.128	4.22 ± 1.045	3.66 ± 1.164	23.98 ± 5.996		
	*F* or *t* value	10.706	5.634	5.357	7.940	2.127	1.898	1.675			
	*P* value	<0.001	<0.001	<0.001	<0.001	0.059	0.091	0.137			

Marital status	Single	3.75 ± 1.115	3.60 ± 1.069	3.71 ± 1.081	3.52 ± 1.141	3.45 ± 1.143	4.28 ± 0.958	3.88 ± 1.045	26.18 ± 5.323	9.806	<0.001
	Married	3.59 ± 1.133	3.50 ± 1.085	3.56 ± 1.082	3.36 ± 1.150	3.40 ± 1.129	4.26 ± 0.954	3.85 ± 1.060	25.51 ± 5.342		
	Divorced	3.61 ± 1.095	3.50 ± 1.016	3.38 ± 1.130	3.42 ± 1.202	3.28 ± 1.226	4.09 ± 1.069	3.59 ± 1.250	24.86 ± 5.639		
	Widowed	3.22 ± 1.222	2.92 ± 1.079	3.36 ± 1.099	3.06 ± 1.120	2.97 ± 1.082	3.97 ± 1.055	3.67 ± 1.095	23.17 ± 5.532		
	*F* or *t* value	9.034	7.139	9.440	8.794	3.222	2.378	2.848			
	*P* value	<0.001	<0.001	<0.001	<0.001	0.022	0.068	0.036			

Education	≤Junior high school	3.49 ± 1.173	3.44 ± 1.119	3.47 ± 1.142	3.29 ± 1.182	3.32 ± 1.187	4.17 ± 0.979	3.71 ± 1.133	24.89 ± 5.672	13.298	<0.001
	High school or secondary school	3.69 ± 1.091	3.59 ± 1.041	3.66 ± 1.063	3.48 ± 1.117	3.44 ± 1.113	4.25 ± 0.961	3.87 ± 1.036	25.98 ± 5.159		
	University or college	3.74 ± 1.122	3.57 ± 1.081	3.69 ± 1.067	3.49 ± 1.143	3.45 ± 1.129	4.31 ± 0.944	3.92 ± 1.025	26.17 ± 5.259		
	≥Postgraduate	3.77 ± 1.054	3.58 ± 1.025	3.66 ± 1.068	3.55 ± 1.273	3.43 ± 1.174	4.41 ± 1.035	3.89 ± 1.108	26.29 ± 5.800		
	*F* or *t* value	11.819	4.031	8.983	7.757	3.260	5.298	8.610			
	*P* value	<0.001	0.007	<0.001	<0.001	0.021	0.001	<0.001			

Permanent residence	Town	3.68 ± 1.113	3.55 ± 1.073	3.63 ± 1.062	3.43 ± 1.144	3.42 ± 1.142	4.32 ± 0.922	3.95 ± 1.016	25.97 ± 5.141	2.061	0.039
	Rural	3.65 ± 1.145	3.55 ± 1.083	3.63 ± 1.116	3.45 ± 1.157	3.42 ± 1.135	4.19 ± 1.004	3.74 ± 1.099	25.64 ± 5.620		
	*F* or *t* value	0.819	-0.211	-0.011	-0.503	0.052	4.337	6.374			
	*P* value	0.413	0.833	0.991	0.615	0.958	<0.001	<0.001			

Occupation	Unemployed	3.56 ± 1.132	3.46 ± 1.088	3.52 ± 1.116	3.32 ± 1.159	3.34 ± 1.148	4.21 ± 0.981	3.76 ± 1.094	25.17 ± 5.408	19.487	<0.001
	Worker	3.72 ± 1.113	3.58 ± 1.052	3.65 ± 1.039	3.49 ± 1.140	3.49 ± 1.132	4.28 ± 0.958	3.95 ± 1.031	26.17 ± 5.354		
	Student	3.74 ± 1.124	3.61 ± 1.078	3.73 ± 1.077	3.53 ± 1.136	3.44 ± 1.131	4.29 ± 0.942	3.89 ± 1.033	26.23 ± 5.251		
	*F* or *t* value	11.680	9.484	15.541	14.954	6.781	3.184	11.784			
	*P* value	<0.001	<0.001	<0.001	<0.001	0.001	0.042	<0.001			

Per capita income in a family (RMB)	≤500	3.62 ± 1.121	3.45 ± 0.999	3.49 ± 1.064	3.34 ± 1.079	3.35 ± 1.080	4.06 ± 0.994	3.58 ± 1.092	24.89 ± 5.223	4.982	<0.001
	501~1500	3.62 ± 1.137	3.54 ± 1.101	3.65 ± 1.100	3.45 ± 1.146	3.39 ± 1.143	4.24 ± 1.011	3.83 ± 1.090	25.72 ± 5.595		
	1501~4000	3.69 ± 1.125	3.54 ± 1.080	3.63 ± 1.088	3.47 ± 1.167	3.45 ± 1.147	4.33 ± 0.916	3.90 ± 1.042	26.03 ± 5.296		
	4001~6500	3.68 ± 1.121	3.57 ± 1.078	3.63 ± 1.090	3.41 ± 1.137	3.40 ± 1.145	4.25 ± 0.937	3.92 ± 1.033	25.86 ± 5.247		
	6501~10000	3.77 ± 1.162	3.71 ± 1.090	3.79 ± 1.081	3.48 ± 1.207	3.47 ± 1.177	4.34 ± 0.937	3.95 ± 1.011	26.52 ± 5.242		
	>10000	3.56 ± 1.022	3.45 ± 1.030	3.50 ± 0.958	3.40 ± 1.067	3.38 ± 1.052	3.99 ± 1.083	3.70 ± 1.033	24.96 ± 5.267		
	*F* or *t* value	1.382	2.607	3.419	1.218	0.954	8.332	8.380			
	*P* value	0.228	0.023	0.004	0.298	0.445	<0.001	<0.001			

Engel's coefficient (%)	≥60	3.52 ± 1.088	3.52 ± 1.091	3.67 ± 1.102	3.48 ± 1.171	3.46 ± 1.087	4.21 ± 0.979	3.83 ± 1.140	25.70 ± 5.415	6.631	<0.001
	50~59	3.64 ± 1.186	3.57 ± 1.102	3.66 ± 1.100	3.49 ± 1.171	3.37 ± 1.145	4.31 ± 0.920	3.94 ± 1.068	25.98 ± 5.518		
	40~49	3.78 ± 1.099	3.65 ± 1.053	3.71 ± 1.055	3.52 ± 1.113	3.46 ± 1.137	4.23 ± 0.999	3.87 ± 1.065	26.22 ± 5.187		
	30~39	3.71 ± 1.111	3.52 ± 1.081	3.60 ± 1.090	3.39 ± 1.162	3.44 ± 1.160	4.30 ± 0.914	3.85 ± 1.023	25.81 ± 5.313		
	<30	3.48 ± 1.130	3.38 ± 1.066	3.47 ± 1.100	3.32 ± 1.155	3.33 ± 1.119	4.22 ± 0.991	3.77 ± 1.051	24.97 ± 5.475		
	*F* or *t* value	9.979	7.557	6.752	4.481	2.007	1.788	2.499			
	*P* value	<0.001	<0.001	<0.001	0.001	0.091	0.128	0.041			

Significant level: *P* < 0.05.

**Table 3:** Multiple stepwise linear regression analysis on the consumers' concern on dairy product label information.

Variables	Category	*β*	SE	*β*′	*t* value	*P* value
Age (y)		-0.271	0.067	-0.066	-4.052	<0.001
	≤20	0.001	0.001	0.001	0.001	<0.001
	21~30	0.199	0.205	0.017	0.971	0.331
	31~40	-0.358	0.252	-0.024	-1.421	0.155
	41~50	-0.447	0.257	-0.030	-1.738	0.082
	51~60	-0.832	0.434	-0.031	-1.918	0.055
	>60	-1.543	0.603	-0.040	-2.560	0.011

Education		0.445	0.105	0.068	4.233	<0.001
	≤Junior high school	-0.869	0.230	-0.067	-3.776	<0.001
	High school or secondary school	-0.012	0.195	-0.001	-0.064	0.949
	University or college	-0.001	0.001	-0.001	-0.001	<0.001
	≥Postgraduate	0.125	0.569	0.003	0.220	0.826

Engel's coefficient (%)		-0.211	0.071	-0.045	-2.983	0.003
	≥60	-0.272	0.340	-0.013	-0.798	0.425
	50~59	-0.120	0.240	-0.008	-0.499	0.618
	40~49	-0.001	0.001	-0.001	-0.001	<0.001
	30~39	-0.328	0.209	-0.027	-1.569	0.117
	<30	-1.068	0.245	-0.074	-4.350	<0.001

Significant level: *P* < 0.05.

## Data Availability

Data from national food risk surveys are funded by the project.
